# Customizing Pore System in a Microporous Metal–Organic Framework for Efficient C_2_H_2_ Separation from CO_2_ and C_2_H_4_

**DOI:** 10.3390/molecules27185929

**Published:** 2022-09-12

**Authors:** Qiang Zhang, Guan-Nan Han, Xin Lian, Shan-Qing Yang, Tong-Liang Hu

**Affiliations:** School of Materials Science and Engineering, National Institute for Advanced Materials, Nankai University, Tianjin 300350, China

**Keywords:** metal–organic frameworks, moderate pore size, hydrogen bond receptor, C_2_H_2_/CO_2_ separation, C_2_H_4_ purification

## Abstract

Selective-adsorption separation is an energy-efficient technology for the capture of acetylene (C_2_H_2_) from carbon dioxide (CO_2_) and ethylene (C_2_H_4_). However, it remains a critical challenge to effectively recognize C_2_H_2_ among CO_2_ and C_2_H_4_, owing to their analogous molecule sizes and physical properties. Herein, we report a new microporous metal–organic framework (**NUM-14**) possessing a carefully tailored pore system containing moderate pore size and nitro-functionalized channel surface for efficient separation of C_2_H_2_ from CO_2_ and C_2_H_4_. The activated **NUM-14** (namely **NUM-14a**) exhibits sufficient pore space to acquire excellent C_2_H_2_ loading capacity (4.44 mmol g^−1^) under ambient conditions. In addition, it possesses dense nitro groups, acting as hydrogen bond acceptors, to selectively identify C_2_H_2_ molecules rather than CO_2_ and C_2_H_4_. The breakthrough experiments demonstrate the good actual separation ability of **NUM-14a** for C_2_H_2_/CO_2_ and C_2_H_2_/C_2_H_4_ mixtures. Furthermore, Grand Canonical Monte Carlo simulations indicate that the pore surface of the **NUM-14a** has a stronger affinity to preferentially bind C_2_H_2_ over CO_2_ and C_2_H_4_ via stronger C-H···O hydrogen bond interactions. This article provides some insights into customizing pore systems with desirable pore sizes and modifying groups in terms of MOF materials toward the capture of C_2_H_2_ from CO_2_ and C_2_H_4_ to promote the development of more MOF materials with excellent properties for gas adsorption and separation.

## 1. Introduction

Accompanied by worldwide economic advances, energy demand and supply have appeared cumulatively prominent; the thirst for high-purity raw materials, conveniently feasible technological streams, and desired final products has turned into even more unprecedented impendency. Industrial chemical separations occupy a large proportion of the quotient of global energy consumption, which has achieved a spectacular 10–15%, amounting to half of the usage amount of industrial energy in the United States [1]. Among the multitudinous commodity chemicals involving industrial interest, acetylene (C_2_H_2_) and ethylene (C_2_H_4_) are the two kinds of the most critical feedstocks for the electronic industry and polymerization manufacturing. Mature C_2_H_2_ fabrication methods in the petrochemical industry mainly rely on thermal cracking of hydrocarbons or partial oxidation of natural gas, and then obtained C_2_H_2_ production can be employed to cut/weld metals or manufacture diversiform high-value chemicals, such as vinyl chloride, acetaldehyde, acrylic acid derivatives, and synthetic fiber/rubber [2]. However, carbon dioxide (CO_2_) as an unwished impurity is inevitably mingled in the obtained C_2_H_2_ production during the aforementioned preparation processes, which will ultimately injure the quality of the downstream chemical products [3]. Therefore, the separation of C_2_H_2_/CO_2_ mixtures became of great importance in the petrochemical industry. In addition, C_2_H_4_, as an indispensable building block in the chemical synthesis industry with global annual production about 201 Mt by 2020, is generally used to manufacture polyethylene, vinyl chloride, ethylene oxide, etc. [4]. C_2_H_4_ is usually obtained by the catalytic cracking of hydrocarbons and steam cracking of naphtha; nevertheless, trace amounts of C_2_H_2_ as contaminants often inevitably coexist in C_2_H_4_ production. These C_2_H_2_ impurities about 1000–5000 ppm not only would significantly affect the quality of the resulting polyethylene but also can further form solid metal acetylide to block the fluid stream-triggering explosion [5]. Under this background, capturing trace C_2_H_2_ from C_2_H_2_/C_2_H_4_ mixture to purify C_2_H_4_ also became an imperative task. The similar geometric characteristics, including molecular size and shape, and alike physical properties mainly referring to the boiling point between C_2_H_2_ and CO_2_ or C_2_H_4_ molecules, render the separations of C_2_H_2_/CO_2_ and C_2_H_2_/C_2_H_4_ mixtures as high-challenge scientific problems [6,7,8]. Researchers in the petrochemical industry have devoted massive efforts to pursuing solutions for overcoming these separation difficulties. The current commercial pathway principally depends on cryogenic distillation, which has a high energy penalty and expensive economic cost. Porous materials utilizing selective physisorption with high energy efficiency, low investment cost, and environmentally friendly peculiarity might provide a promising alternative to separate these troublesome mixtures.

Metal–organic frameworks (MOFs) with large surface area, high porosity, fascinating modularity, and abundant functionality, as new-style crystalline porous materials, are constructed with metal ions/clusters and organic linkers. Because of their unique characteristics, MOFs have received wide attention in numerous application fields, such as gas storage and separation, heterogeneous catalysis, proton conduction, and fluorescence detection [9,10,11,12]. The most remarkable advantage of MOF materials over other traditional porous materials (such as zeolite, silica gel, and porous carbon) lies in the tunability of the pore system, predominantly referring to pore size/shape and pore surface chemistry. Plentiful adsorption-based MOFs were explored to address C_2_H_2_ separations challenges, which have obtained certain achievements [13,14]. Although a large number of research results indicated that incorporating strong binding sites (such as Lewis basic sites and open metal sites) into large-pore MOFs can effectively promote high C_2_H_2_ uptake capacities, most of them exhibited low separation selectivity and even terrible actual dynamic breakthrough properties due to their large pore size [15,16]. On the other side, the introduction of fluorinated anions (such as SiF_6_^2−^, NbOF_5_^2−^, and TiF_6_^2−^) as hydrogen-bonding acceptors (HBAs) into ultra-microporous pillared hybrid MOFs can preferentially bind C_2_H_2_ molecules to dramatically enhance the separation selectivity [17,18]. However, the majority of ultra-microporous MOFs with excellent separation performance exhibited relatively low C_2_H_2_ loading capacity owing to their limited pore space. Recently, through the structure–performance relationship screening of 62 reported top-performing C_2_H_2_ adsorbents, Zhai et al. found that tuning the aperture of MOFs within the range of moderate pore size (5.0–7.5 Å), combined with accessible HBAs, is an effective route to break through the trade-off barrier between C_2_H_2_ storage and C_2_H_2_/CO_2_ separation [19]. Many studies have shown that embedding functional groups (such as -F, -NO_2_, -NH_2_, -OH) into MOF channels can also improve the separation selectivity of C_2_H_2_/C_2_H_4_ without a slight sacrifice in intrinsic moderate pore volumes or surface areas to adsorb a large amount of the objective gas molecules [20,21]. Therefore, if a porous MOF contains a suitable pore system that is simultaneously provided with both moderate pore size and high-density accessible functional groups like HBAs, it may synchronously possess satisfactory separation performance for C_2_H_2_/CO_2_ and C_2_H_2_/C_2_H_4_ mixtures. Although multiple design strategies, such as the isoreticular chemistry principle [22], pore–space partition strategy [23], and forming structural interpenetration [24], have been developed to guide the synthesis of desired MOF materials, difficult challenges still remain in customizing MOF materials with desirable pore systems. This is because even subtle effects can lead to undesirable results in the uncontrollable and complex self-assembly process of the MOFs.

Herein, we designed and synthesized a novel Ni-MOF {[Ni(TPT)(NPC)(H_2_O)]·solvent}_n_ (**NUM-14**, TPT = 2,4,6-tri(4-pyridinyl)-1,3,5-triazine, H_2_NPC = 3-nitrophthalic acid), featuring a desired pore size of 5.8 Å and functionalized pore environment decorated by abundant nitro groups serving as hydrogen bond receptors, which can preferentially capture C_2_H_2_ for achieving efficient separation of C_2_H_2_/CO_2_ and C_2_H_2_/C_2_H_4_ mixtures. The single-component adsorption isotherms indicate that **NUM-14a** (activated **NUM-14**) has a good adsorption capacity for C_2_H_2_ with 4.44 mmol g^−1^, exceeding some cutting-edge MOF materials. The predicted IAST selectivities and practical breakthrough experiments for C_2_H_2_/C_2_H_4_ and C_2_H_2_/CO_2_ mixtures demonstrated that **NUM-14a** has favourable separation potential for these two kinds of mixed gases. Moreover, the values of *Q*_st_ calculation quantitatively vindicated that **NUM-14a** owns the strongest interaction with C_2_H_2,_ stronger than with CO_2_ and C_2_H_4_, which is also proved from another side by Grand Canonical Monte Carlo (GCMC) calculations. Both experimental and theoretical results show that **NUM-14a** can realize the efficient separation of C_2_H_2_ from CO_2_ and C_2_H_4_, and that this fine performance mainly comes from its suitable pore system.

## 2. Results and Discussion

### 2.1. Single Crystal X-ray Diffraction Structure

**Figure 1:**
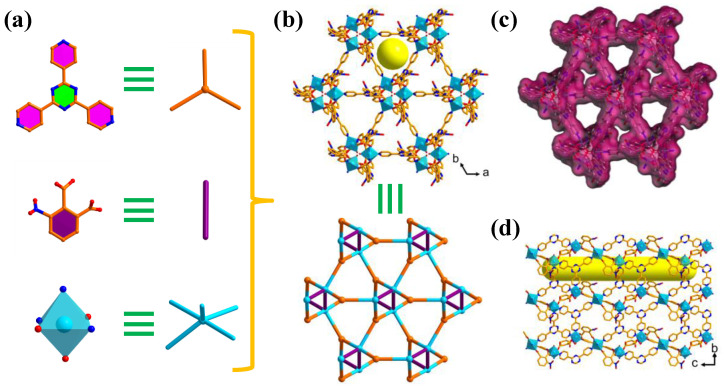
(**a**) Topology simplification of NiN_3_O_3_ octahedron and the ligands. (**b**) The triangular channel structure and topology simplification of 3D framework in **NUM-14** along the c axis. (**c**) The Connolly surface void spaces of **NUM-14**. (**d**) The side view of channel structure in **NUM-14** along the a-axis. Color code: Ni, sky blue; O, red; N, blue; C, light orange. Guest molecules and H atoms have been omitted for clarity.

The solvothermal reaction of TPT, H_2_NPC, and Ni(NO_3_)_2_ in a DMF/H_2_O mixed solvent system at 100 °C for 72 h harvested jade-green blocky crystals of as-synthesized **NUM-14**. The crystallographic structure analysis manifested that **NUM-14** crystallizes in the trigonal *P*3_1_21 space group. The asymmetric unit of **NUM-14** contains one Ni^2+^ atom, half of deprotonated NPC^2−^ ligand, and a half of TPT ligand. Each Ni^2+^ atom has a six-coordinate mode to form slightly distorted octahedral geometry with three N atoms from three different TPT ligands and two carboxylate O atoms from two different NPC^2−^ linkers, as well as one terminal water molecule. Each NPC^2−^ ligand connected two Ni^2+^ atoms to form a 1D infinite helix chain spiraling counterclockwise along the c axis. Each TPT ligand also coordinate with two Ni^2+^ atoms to generate two kinds of infinite 1D helix chains along the c axis, in which one of them rotates clockwise and another inversely rotates counterclockwise (Figure S1). Then three types of spiral chains make up one channel column. The remaining end of TPT ligands as pore wall on each channel column is connected to the adjacent channel column to form two kinds of one-dimensional channel structures along the c axis, one of which is the triangular channel with an aperture of about 6 Å while the another is similar to the former except that the nitro groups on the NPC^2−^ linkers toward its interior (Figure 1b–d). The coordinate mononuclear Ni^2+^ octahedron and TPT ligand can be simplified as 5- and 3-connected nodes, respectively (Figure 1a). Therefore, **NUM-14** can be simplified as a 2-nodal 3,5-connected topology network with the point symbol of (4·7^2^)(4^3^·6^2^·7^4^·8) (Figure 1b) [25]. Calculation by *PLATON* revealed that the solvent-accessible volume in fully desolvated **NUM-14** is 59.4%. The accessible channel surface in **NUM-14a** is mainly composed of the pyridine/triazine rings and abundant nitro groups in ligands, getting a very polar and rich hydrogen bond receptor pore environment.

### 2.2. Purity and Stabilities of NUM-14

The experimental and activated PXRD patterns are strongly consistent with the simulated model based on the single-crystal data confirming the high-phase purity and skeleton stability after the degassing of **NUM-14** (Figure S2). The thermostability was demonstrated by TGA. As shown in Figure S3, it can be seen that the skeleton of **NUM-14** remains stable up to 300 °C. As shown in Figure S4, through testing PXRD of **NUM-14** samples soaked in various common solvents for 1 week, we found that **NUM-14** acquits itself well in solvent stability under different conditions.

### 2.3. Gas Adsorption Properties of NUM-14a

**Figure 2:**
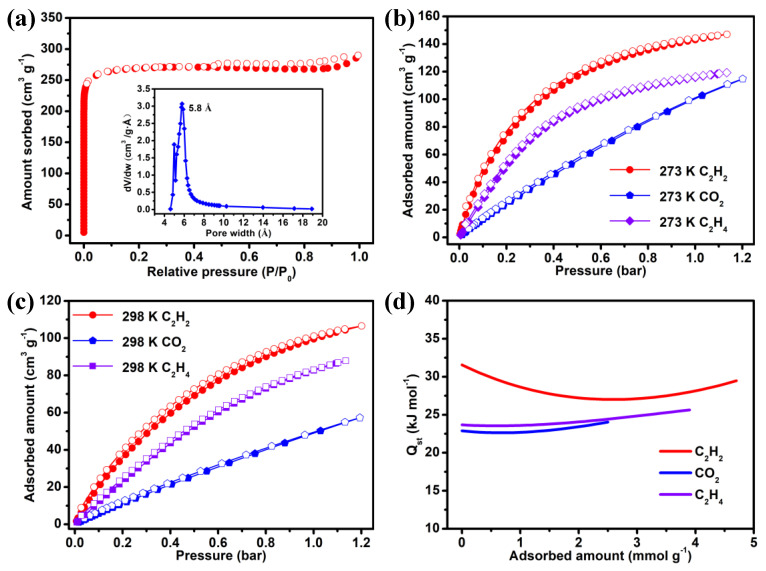
(**a**) N_2_ sorption isotherm and pore size distribution of **NUM-14a** at 77 K. Single-component gas isotherms of C_2_H_2_, CO_2_, and C_2_H_4_ for **NUM-14a** at (**b**) 273 K and (**c**) 298 K (filled and open symbols represent adsorption and desorption curves, respectively). (**d**) Isosteric enthalpy of adsorption of C_2_H_2_, CO_2_, and C_2_H in **NUM-14a**.

The activated sample of **NUM-14a** was prepared by heating the CH_2_Cl_2_-exchanged sample under preset high vacuum conditions at 40 °C for the duration of 10 h. Then, the N_2_ sorption isotherm was recorded at 77 K to characterize and evaluate the permanent porosity of **NUM-14a**. As indicated in Figure 2a, it shows a reversible type-I adsorption curve with the saturated loading of 289.9 cm^3^ g^−1^ and is akin to multitudinous typical microporous MOF materials [26,27]. According to the N_2_ adsorption isotherm, the BET (Brunauer–Emmett–Teller) and Langmuir surface areas were calculated, which reached 1075.5 and 1194.2 m^2^ g^−1^, respectively. The Horvath–Kawazoe method was applied to calculate the pore size distribution and the result exhibited the main aperture concentrates upon 5.8 Å, which allows small gas molecules to pass through easily.

Combining with appropriate pore size and functionalized polar surface, **NUM-14a** is promising for evaluating C_2_H_2_ adsorption and separation performance from CO_2_ and C_2_H_4_. As shown in Figure 2b,c, single-component sorption isotherms of **NUM-14a** for C_2_H_2_, CO_2_, and C_2_H_4_ were measured at 273 and 298 K. Under 1 bar, these pure-component gas adsorption isotherms revealed that the C_2_H_2_ adsorption capacities of **NUM-14a** are 143.1 and 99.5 cm^3^ g^−1^ at 273 and 298 K, respectively, which is markedly higher than that of CO_2_ (102 and 50.2 cm^3^ g^−1^) and C_2_H_4_ (115.8 and 82.7 cm^3^ g^−1^), implying the distinctly stronger adsorption ability of **NUM-14a** for C_2_H_2_ than CO_2_ and C_2_H_4_. Noteworthily, the adsorption capacity of C_2_H_2_ (4.44 mmol g^−1^) at 298 K and 1 bar in **NUM-14a** is superior to most top-performing MOFs, like Zn-FBA (1.03 mmol g^−1^) [28], IPM-101 (2.55 mmol g^−1^) [29], ZNU-1 (3.41 mmol g^−1^) [30], ZNU-4 (3.58 mmol g^−1^) [31], and ZJU-74a (3.83 mmol g^−1^) [32], which is attributed to its high porosity. Moreover, the C_2_H_2_/CO_2_ uptake ratio at 298 K and 1 bar for **NUM-14a** reaches 1.98, while the uptake ratio of C_2_H_2_/C_2_H_4_ is only 1.20. Inspired by the distinctive uptake capacity and preferential binding of C_2_H_2_ for **NUM-14a**, we then calculated the isosteric enthalpies of adsorption (*Q*_st_), a crucial metric that quantifies the interaction strength between gas molecules and pores in MOFs, by the Virial-type equation to quantitatively estimate the binding affinity between gas molecules and host framework (Figures S5–S7). The calculated *Q*_st_ at near-zero coverage for C_2_H_2_ (31.57 kJ mol^−1^) is higher than CO_2_ (22.89 kJ mol^−1^) and C_2_H_4_ (23.67 kJ mol^−1^), demonstrating the relatively stronger host–guest affinity for **NUM-14a** toward C_2_H_2_ in contrast to CO_2_ and C_2_H_4_ (Figure 2d). These results of *Q*_st_ are adequately consistent with the gas adsorption behaviors as depicted in single-component isotherms and prove the feasibility of the C_2_H_2_ preferential adsorption in the framework of **NUM-14a**.

### 2.4. Gas Separation Performances of NUM-14a

**Figure 3:**
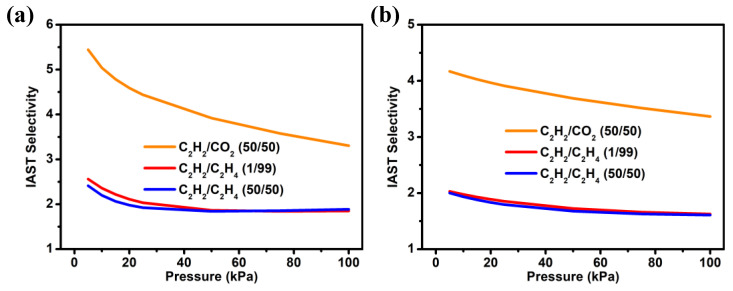
(**a**) The adsorption selectivities of **NUM-14a**, predicted from IAST for C_2_H_2_/CO_2_ (50:50, *v*/*v*), C_2_H_2_/C_2_H_4_ (1:99, *v*/*v*) and C_2_H_2_/C_2_H_4_ (50:50, *v*/*v*) at 273 K (**a**) and 298 K (**b**).

Given the preferential capture and stronger affinity of C_2_H_2_ than CO_2_ and C_2_H_4_ in **NUM-14a**, IAST was further adopted to evaluate the separation performances of **NUM-14a** for C_2_H_2_/CO_2_ and C_2_H_2_/C_2_H_4_ mixtures. Single-component adsorption isotherms of C_2_H_2_ and C_2_H_4_ obtained from experimental determinations were fitted by the dual-site Langmuir–Freundlich model, while the isotherms of CO_2_ were fitted by the single-site Langmuir–Freundlich equation for pursuing the more accurate consistency between experimental data and theoretical model (Figures S8–S13). The fitting results were then used to predictably calculate adsorptive selectivities for equimolar C_2_H_2_/CO_2_ and C_2_H_2_/C_2_H_4_ with two different ratios of 50/50 or 1/99 at 273 and 298 K (Figure 3a,b). As revealed in Figure 3b, the calculated selectivity of **NUM-14a** for equimolar C_2_H_2_/CO_2_ (50:50, *v*/*v*) is 3.37 at 298 K and 100 kPa, which is lower than some famous MOF materials under the same conditions, such as CuI@UiO-66-(COOH)_2_ (185.00) [33], ATC-Cu (53.60) [34], and MOF-OH (25.00) [35], and slightly lower than that of FJU-118a (7.80) [36], BUT-85 (6.10) [37], but higher than many other well-known materials, such as [Ca(dtztp)_0.5_] (1.70) [38], [Ni(tzba)_0.5_(F)(bpy)] (2.20) [39], CAU-10H (2.50) [40] and SNNU-5-Sc (2.66) [41]. Due to the similar C_2_H_2_ and C_2_H_4_ adsorption behavior, although **NUM-14a** exhibits relatively low selectivities for C_2_H_2_/C_2_H_4_ mixtures with volume ratios of 1:99 (1.63) and 50:50 (1.61) at 298 K and 100 kPa, the result for C_2_H_2_/C_2_H_4_ (1:99, *v*/*v*) is still comparable to many consequences of reported MOFs, such as 1.13 for NUM-12a [42], 1.61 for Zn(ad)(int) [4], 1.59 for ZJNU-14 [43], 1.77 for ZJNU-7 [44], and 2.1 for UiO-67-(NH_2_)_2_ (Table S2) [45].

**Figure 4:**
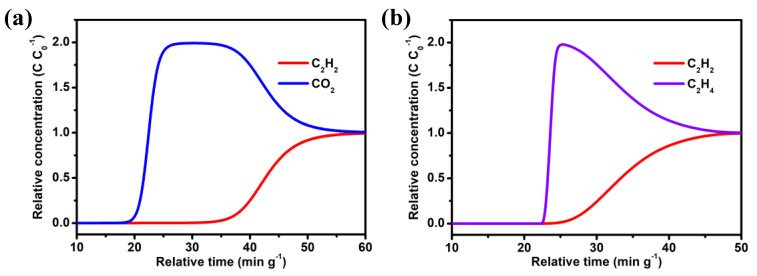
The column breakthrough curves for mixture gases of (**a**) C_2_H_2_/CO_2_ (50:50, *v*/*v*), and (**b**) C_2_H_2_/C_2_H_4_ (50:50, *v*/*v*). The experiments were conducted at 298 K and the inlet gas flow rate was maintained at 2 mL min^−1^.

To further evaluate the separation performance of **NUM-14a** for C_2_H_2_/CO_2_ and C_2_H_2_/C_2_H_4_ mixtures, we performed dynamic breakthrough experiments in a packed tube using **NUM-14a** as physical adsorbent at 298 K under a total inlet gas flow rate of 2 mL min^−1^. As shown in Figure 4a, the breakthrough curve clearly proves that **NUM-14a** can effectively separate the C_2_H_2_/CO_2_ mixture. When the equimolar C_2_H_2_/CO_2_ gas mixture passes through the adsorption column, the CO_2_ gas first elutes due to its deficient uptake capacity. Then, C_2_H_2_ breaks through the packed column with a penetration time of 19 min g^−1^ and the adsorbent slowly tends to saturate soon afterward. Ultimately, the outlet gas flow reaches adsorption equilibrium with the same components as the imported stream at 55 min g^−1^. Similarly, the separation performance of **NUM-14a** for the equimolar C_2_H_2_/C_2_H_4_ mixture is described in Figure 4b. The experiment result displays that C_2_H_2_ gas can be more preferably adsorbed in the separation unit than C_2_H_4_, so the pure C_2_H_4_ can be obtained with a time interval of 5 min g^−1^, which is shorter than the breakthrough time of the C_2_H_2_/CO_2_ mixture because of the stronger adsorption capacity of C_2_H_4_ relative to CO_2_. These breakthrough tests undoubtedly testify to the ability of **NUM-14a** for separating C_2_H_4_ from mixtures containing CO_2_ or C_2_H_4_.

### 2.5. Adsorption Mechanism

**Figure 5:**
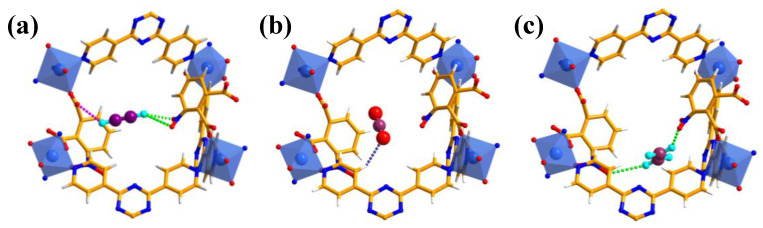
The GCMC calculated adsorption sites of C_2_H_2_ (**a**), CO_2_ (**b**), and C_2_H_4_ (**c**) in **NUM-14a** at 298 K and 1 bar.

To profoundly investigate the interesting gas adsorption behaviors between the preferential sites and adsorbed gas molecules within **NUM-14a**, GCMC calculations were conducted to probe the interaction between the gas molecules and the host framework at 298 K and 1 bar. As shown in Figure 5a, for adsorbed C_2_H_2_ in pores, three strong binding sites of hydrogen bonds were found, where two distances of C-H···O between C_2_H_2_ and two nitro oxygen atoms are 2.378 Å and 3.608 Å, and the distance of C-H···O between terminal hydrogen atom of C_2_H_2_ and one carbonyl oxygen atom is 2.611 Å. The CO_2_ molecule only interacts with the framework of **NUM-14a** through one weak C-H···O (3.562 Å) hydrogen bond interaction between acidic hydrogen atom on the pyridine ring and the basic oxygen atom at the end of CO_2_ molecule (Figure 5b). For the C_2_H_4_ molecule which has weaker acidic than C_2_H_2_ and therefore has a weaker affinity to basic sites, two hydrogen bond interactions between C_2_H_4_ with two nitroxides were sought with long C-H···O distances (2.487 and 3.074 Å) (Figure 5c). By comparing GCMC calculation results, we can find that the pore surface of **NUM-14a** shows a stronger recognition effect on C_2_H_2_ molecules than CO_2_ and C_2_H_4_, explaining the reason for outstanding capture and separation performance of C_2_H_2_ in **NUM-14a** observed in experiments.

According to the adsorption data, we can find that **NUM-14a** has a good adsorption capacity for C_2_H_2_, which is mainly attributed to its medium pore size and large porosity. Moreover, the combination of separation experiments and the simulation calculations shows that **NUM-14a** has stronger binding forces for C_2_H_2_ compared with CO_2_ and C_2_H_4_, which is possibly ascribed to the stronger synergistic hydrogen bond interactions between C_2_H_2_ and nitro group and the exposed oxygen atom. All of these prove that the customized fabrication of a pore system with moderate pore size and modified surface with HBAs in MOFs is beneficial to separate C_2_H_2_/CO_2_ and C_2_H_2_/C_2_H_4_ mixtures.

## 3. Materials and Methods

### 3.1. Materials and Characterization

All chemicals and reagents were purchased from commercial suppliers and used without further purification. Powder X-ray diffraction (PXRD) was measured on a Rigaku Miniflex 600 with Cu Kα radiation (λ = 1.5425 Å) under air conditions. Thermogravimetric analysis (TGA) was recorded on a Rigaku standard thermogravimetry-differential thermal analysis (TG-DTA) analyzer, utilizing an empty and clean Al_2_O_3_ crucible as reference (heating rate = 10 °C min^−1^ in Ar atmosphere).

### 3.2. Gas Sorption Measurements

Before the sorption measurement, the sample of **NUM-14** was soaked in dichloromethane for 3 days to exchange solvent molecules in the channels. The degas procedure for the solvent-exchanged **NUM-14** was conducted at 40 °C under a high vacuum (less than 10^−5^ Torr) for 10 h and led to the formation of activated sample **NUM-14a**. The N_2_ sorption isotherm measurement was carried out using a Micrometrics ASAP 2460 volumetric gas adsorption analyzer at 77 K in a liquid nitrogen bath. The C_2_H_2_, C_2_H_4_, and CO_2_ sorption isotherm measurements were carried out at 273 and 298 K respectively using a Micrometrics ASAP 2020M volumetric gas adsorption analyzer.

### 3.3. X-ray Crystallography

Single-crystal X-ray diffraction data of **NUM-14** were collected on the Rigaku XtaLAB PRO MM007 DW diffractometer with Cu Kα radiation (λ = 1.54184 Å) at T = 99.99 (1) K. The structure was solved with the *SHELXT* program and refined by full-matrix least-squares against F^2^ using the *SHELXL* program [46,47]. Anisotropic thermal parameters were implemented to all non-hydrogen atoms, and all hydrogen atoms were placed in the calculated positions and refined with isotropic thermal parameters. The *Solvent Mask* in *Olex2* software was employed to remove scattering contributions of the disordered solvent molecules and the generated solvent-free data of direction intensities were further refined [48]. Details of the crystal parameters, data collection, and refinement of **NUM-14** are listed in Table S1.

### 3.4. Synthesis of NUM-14

A solvothermal reaction of Ni(NO_3_)_2_·6H_2_O (29.0 mg, 0.1 mmol), TPT (18.7 mg, 0.06 mmol), and H_2_NPC (21.1 mg, 0.1 mmol) in 2.5 mL of a mixed solvent of DMF/H_2_O (4:1, *v*/*v*) was kept at 100 °C for 3 days, and blocky crystals of **NUM-14** were first time synthesized in 83% yield based on the TPT ligand. When the reaction temperature slowly cooled to room temperature, a fresh sample was collected by filtration and washed with fresh DMF several times.

### 3.5. Isosteric Enthalpy of Adsorption Calculations

The experimental adsorption data of C_2_H_2_, CO_2_, and C_2_H_4_ at 273 and 298 K in **NUM-14a** were fitted using a virial model (Equation (1)):

(1) $$\ln P=\ln N+\frac{1}{T}\overset{m}{\underset{i=0}{\sum }}a_{i}N^{i}+\overset{n}{\underset{j=0}{\sum }}b_{j}N^{j}$$

where ***P*** is the pressure in Torr, ***N*** is the adsorbed amount in mmol g^−1^, ***T*** is the temperature in K, and ***a_i_*** and ***b_j_*** are virial coefficients.

The isosteric enthalpies of adsorption (***Q_st_***) were calculated based on the fitted virial coefficients using the following equation (Equation (2)):

(2) $$Q_{st}=-R\overset{m}{\underset{i=0}{\sum }}a_{i}N^{i}$$

***Q_st_*** is the coverage-dependent isosteric heat of adsorption and ***R*** is the universal gas constant.

### 3.6. Adsorption Selectivity Calculations

Ideal adsorbed solution theory (IAST) was utilized to predict gas adsorption selectivity of binary mixtures from the experimental single-component isotherms of C_2_H_2_, CO_2_, and C_2_H_4_ [49]. The experimental pure-component adsorption isotherms of C_2_H_2_ and C_2_H_4_ were initially fitted by the dual-site Langmuir–Freundlich model (Equation (3)), while CO_2_ was fitted by the single-site Langmuir–Freundlich model. That is, only the first half of Equation (3) is used to fit the experimental data to obtain a perfect fitting degree:

(3) $$q=\frac{q_{A,sat}b_{A}p^{1/n_{1}}}{1+b_{A}p^{1/n_{1}}}\;+\frac{q_{B,sat}b_{A}p^{1/n_{2}}}{1+b_{B}p^{1/n_{2}}}$$

where ***p*** is the pressure in kPa, ***q*** is the adsorbed amount in mmol g^−1^, and ***q_A,sat_*** and ***q_B,sat_*** are the saturation capacities of two distinct adsorption sites ***A*** and ***B*** in mmol g^−1^. ***b_A_*** and ***b_B_*** are the affinity coefficients in kPa^−1^, and ***n_1_*** and ***n_2_*** represent the deviations from an ideal homogeneous surface.

Then the fitted parameters were used to calculate the selectivity (Equation (4)):

(4) $$S_{A/B}=\frac{X_{A}/X_{B}}{Y_{A}/Y_{B}}$$

In which, ***X_i_*** and ***Y_i_*** represent the mole fractions of component ***i*** in the adsorbed and bulk phases, respectively.

### 3.7. Breakthrough Experiments

The breakthrough experiments for C_2_H_2_/CO_2_ (50:50, *v*/*v*) and C_2_H_2_/C_2_H_4_ (50:50, *v*/*v*) mixtures were completed on the Multi-component Adsorption Breakthrough Curve Analyzer from Beishide Instrument Technology Co., Ltd. (Beijing, China). An activated crystalline powder sample (1.30 g) was packed into a breakthrough column (6 mm diameter and 4 mL volume) with a 3 cm length of the sample loading, which was purged with He flow (50 mL min^−1^), sustaining 120 min at 40 °C before each breakthrough experiment. Subsequently, the mixed gas flows of C_2_H_2_/CO_2_ and C_2_H_2_/C_2_H_4_ with a flow rate of 2 mL min^−1^ were respectively introduced into the adsorber at 298 K. At the same time, the compositions of effluents from the packed column were monitored and analyzed in real-time by online mass spectrometry.

### 3.8. Grand Canonical Monte Carlo Simulations

The GCMC simulations were performed for the adsorption of C_2_H_2_, CO_2_, and C_2_H_4_ in **NUM-14a** with the Material Studio 8.0. The optimal adsorption sites were simulated under 298 K with a pressure of 1.0 bar. We used 1.0 × 10^7^ cycles for equilibration, and the production steps were set to 1.0 × 10^6^. The framework of **NUM-14a** and adsorbate molecules were treated as a rigid structure. A standard Lennard–Jones and Coulomb model was used and the Lennard–Jones parameters for the framework atoms as well as adsorbate molecules were adopted from the universal force field (UFF). Ewald summation was used to calculate electrostatic interactions for both adsorbent–adsorbate and adsorbateadsorbate interactions.

## 4. Conclusions

In summary, we constructed a new Ni-based MOF **NUM-14**, which has a fascinating pore system featuring an appropriate pore size and pleasant pore environment, to separate C_2_H_2_ well from CO_2_ and C_2_H_4_. By a feat of its pore characteristic, **NUM-14a** can adsorb more C_2_H_2_ than CO_2_ and C_2_H_4_. With the advantages of a nitro-functionalized pore wall framework, **NUM-14a** expresses greater affinity to C_2_H_2_ through hydrogen bond interactions. Furthermore, the strong pure-component gas adsorption behavior and favorable C_2_H_2_/CO_2_, and C_2_H_2_/C_2_H_4_ separation performance are achieved by **NUM-14a**. In addition, the underlying selective adsorption mechanism and separation reason are revealed by GCMC simulations at the molecular level. This work can guide the designed synthesis of desirable MOFs with a tailor-made pore system and practical application of them for high-challenge gas separation problems.

## Data Availability

The data that support the findings of this study are available in the Supplementary Materials of this article or from the corresponding author upon reasonable request.

## Supplementary Materials

The following supporting information can be downloaded at: https://www.mdpi.com/article/10.3390/molecules27185929/s1. Table S1. Crystal data and structure refinement parameters for **NUM-14**. Table S2. Comparisons of C_2_H_2_ uptake and selectivities of C_2_H_2_/CO_2_ and C_2_H_2_/C_2_H_4_ for **NUM-14****a** and other MOFs. Figure S1. Three kinds of helix chains constitute the channel column of **NUM-14**. Figure S2. Comparison of simulated, experimental, and activated PXRD patterns of **NUM-14**. Figure S3. TGA curve for **NUM-14** under Ar atmosphere. Figure S4. The PXRD patterns for **NUM-14** after immersed in common solvents a week. Figures S5–S7. The details of Virial equation fitting to the experimental C_2_H_2_, CO_2_, and C_2_H_4_ adsorption data for **NUM-14a**. Figures S8–S13. The details of dual/single-site Langmuir–Freundlich isotherm fitting to the experimental C_2_H_2_, CO_2_, and C_2_H_4_ adsorption data for **NUM-14a** at 273 and 298 K. Figures S14–S16. Density distributions of C_2_H_2_, CO_2_, and C_2_H_4_ in **NUM-14a** at 298 K and 1 bar.

## Sample Availability

Samples of the compound **NUM-14** are available from the authors.
