# Supplementary material for: Customizing Pore System in a Microporous Metal–Organic Framework for Efficient C2H2 Separation from CO2 and C2H4
Source: Molecules. 2022 Sep 12;27(18):5929. doi: 10.3390/molecules27185929 (PMC9502222; doi:10.3390/molecules27185929)

checkCIF/PLATON report

Structure factors have been supplied for datablock(s) NUM-14

THIS REPORT IS FOR GUIDANCE ONLY. IF USED AS PART OF A REVIEW PROCEDURE FOR PUBLICATION, IT SHOULD NOT REPLACE THE EXPERTISE OF AN EXPERIENCED CRYSTALLOGRAPHIC REFEREE.

No syntax errors found. CIF dictionary Interpreting this report

Datablock: NUM-14

```
Bond precision:   C-C = 0.0041 A          Wavelength=1.54184

Cell:              a=15.1174(1)      b=15.1174(1)      c=18.8520(2)
                   alpha=90          beta=90           gamma=120
Temperature:       100 K
```

	Calculated	Reported
Volume	3731.15(6)	3731.14(6)
Space group	P 31 2 1	P 31 2 1
Hall group	P 31 2"	P 31 2"
Moiety formula	C26.13 H17.07 N7.02 Ni O7.04 [+ solvent]	C26.129 H17.064 N7.021 Ni O7.043
Sum formula	C26.13 H17.07 N7.02 Ni O7.04 [+ solvent]	C26.13 H17.06 N7.02 Ni O7.04
Mr	600.82	600.77
Dx,g cm-3	0.802	0.802
Z	3	3
Mu (mm-1)	0.842	0.842
F000	922.1	922.0
F000’	915.97	
h,k,lmax	18,18,23	18,18,23
Nref	5033[2811]	4864
Tmin,Tmax	0.817,0.845	0.862,1.000
Tmin’	0.777	

```
Correction method= # Reported T Limits: Tmin=0.862 Tmax=1.000
AbsCorr = MULTI-SCAN

Data completeness= 1.73/0.97        Theta(max)= 73.614
```

R(reflections)= 0.0349(4633)

wR2(reflections)= 0.1010(4864)

S = 1.062

Npar= 231

The following ALERTS were generated. Each ALERT has the format
test-name_ALERT_alert-type_alert-level.
Click on the hyperlinks for more details of the test.

Alert level C

```
PLAT220_ALERT_2_C NonSolvent   Resd 1  N   Ueq(max)/Ueq(min) Range        4.2 Ratio
PLAT220_ALERT_2_C NonSolvent   Resd 1  O   Ueq(max)/Ueq(min) Range        4.0 Ratio
PLAT234_ALERT_4_C Large Hirshfeld Difference O4       --N5       .       0.17 Ang.
PLAT234_ALERT_4_C Large Hirshfeld Difference O5       --N5       .       0.17 Ang.
PLAT911_ALERT_3_C Missing FCF Refl Between Thmin & STh/L=    0.600         10 Report
```

Alert level G

```
PLAT002_ALERT_2_G Number of Distance or Angle Restraints on AtSite          4 Note
PLAT003_ALERT_2_G Number of Uiso or Uij Restrained non-H Atoms ...         11 Report
PLAT004_ALERT_5_G Polymeric Structure Found with Maximum Dimension          3 Info
PLAT007_ALERT_5_G Number of Unrefined Donor-H Atoms .............           2 Report
PLAT012_ALERT_1_G N.O.K.   _shelx_res_checksum Found in CIF ......     Please Check
PLAT033_ALERT_4_G Flack x Value Deviates > 3.0 * sigma from Zero .      0.100 Note
PLAT041_ALERT_1_G Calc. and Reported SumFormula    Strings  Differ     Please Check
PLAT042_ALERT_1_G Calc. and Reported MoietyFormula Strings  Differ     Please Check
PLAT172_ALERT_4_G The CIF-Embedded .res File Contains DFIX Records          3 Report
PLAT178_ALERT_4_G The CIF-Embedded .res File Contains SIMU Records          4 Report
PLAT186_ALERT_4_G The CIF-Embedded .res File Contains ISOR Records          3 Report
PLAT187_ALERT_4_G The CIF-Embedded .res File Contains RIGU Records          1 Report
PLAT230_ALERT_2_G Hirshfeld Test Diff for    O5       --C16_d    .        9.5 s.u.
PLAT230_ALERT_2_G Hirshfeld Test Diff for    N5       --C13      .       11.1 s.u.
PLAT230_ALERT_2_G Hirshfeld Test Diff for    C11      --C12      .       18.5 s.u.
PLAT230_ALERT_2_G Hirshfeld Test Diff for    C11      --C17_d    .        9.2 s.u.
PLAT230_ALERT_2_G Hirshfeld Test Diff for    C17      --C11_d    .        9.2 s.u.
PLAT300_ALERT_4_G Atom Site Occupancy of H1A       Constrained at         0.5 Check
PLAT300_ALERT_4_G Atom Site Occupancy of H1B       Constrained at         0.5 Check
PLAT301_ALERT_3_G Main Residue  Disorder ..............(Resd  1  )        21% Note
PLAT335_ALERT_2_G Check Large C6 Ring C-C Range C12      -C17            0.16 Ang.
PLAT414_ALERT_2_G Short Intra D-H..H-X       H1B      ..H4       .       2.13 Ang.
                                              1+y,-1+x,1-z  =      4_646 Check
PLAT415_ALERT_2_G Short Inter D-H..H-X       H1A      ..H4       .       2.13 Ang.
                                          1-y,-1+x-y,1/3+z  =      2_645 Check
PLAT606_ALERT_4_G Solvent Accessible VOID(S) in Structure ........          ! Info
PLAT773_ALERT_2_G Check long C-C Bond in CIF: C11       --C12            1.74 Ang.
PLAT789_ALERT_4_G Atoms with Negative _atom_site_disorder_group  #         12 Check
PLAT860_ALERT_3_G Number of Least-Squares Restraints ............         117 Note
PLAT912_ALERT_4_G Missing # of FCF Reflections Above STh/L=  0.600         39 Note
PLAT913_ALERT_3_G Missing # of Very Strong Reflections in FCF ....          1 Note
PLAT933_ALERT_2_G Number of HKL-OMIT Records in Embedded .res File          5 Note
PLAT941_ALERT_3_G Average HKL Measurement Multiplicity ..........         4.5 Low
PLAT978_ALERT_2_G Number C-C Bonds with Positive Residual Density.          0 Info
```

---

0 **ALERT level A** = Most likely a serious problem - resolve or explain

```
 0 ALERT level B = A potentially serious problem, consider carefully
 5 ALERT level C = Check. Ensure it is not caused by an omission or oversight
32 ALERT level G = General information/check it is not something unexpected

 3 ALERT type 1 CIF construction/syntax error, inconsistent or missing data
15 ALERT type 2 Indicator that the structure model may be wrong or deficient
 5 ALERT type 3 Indicator that the structure quality may be low
12 ALERT type 4 Improvement, methodology, query or suggestion
 2 ALERT type 5 Informative message, check
```

It is advisable to attempt to resolve as many as possible of the alerts in all categories. Often the minor alerts point to easily fixed oversights, errors and omissions in your CIF or refinement strategy, so attention to these fine details can be worthwhile. In order to resolve some of the more serious problems it may be necessary to carry out additional measurements or structure refinements. However, the purpose of your study may justify the reported deviations and the more serious of these should normally be commented upon in the discussion or experimental section of a paper or in the "special_details" fields of the CIF. checkCIF was carefully designed to identify outliers and unusual parameters, but every test has its limitations and alerts that are not important in a particular case may appear. Conversely, the absence of alerts does not guarantee there are no aspects of the results needing attention. It is up to the individual to critically assess their own results and, if necessary, seek expert advice.

Publication of your CIF in IUCr journals

A basic structural check has been run on your CIF. These basic checks will be run on all CIFs submitted for publication in IUCr journals (*Acta Crystallographica*, *Journal of Applied Crystallography*, *Journal of Synchrotron Radiation*); however, if you intend to submit to *Acta Crystallographica Section C* or *E* or *IUCrData*, you should make sure that full publication checks are run on the final version of your CIF prior to submission.

Publication of your CIF in other journals

Please refer to the *Notes for Authors* of the relevant journal for any special instructions relating to CIF submission.

PLATON version of 18/05/2022; check.def file version of 17/05/2022

Datablock NUM-14 - ellipsoid plot

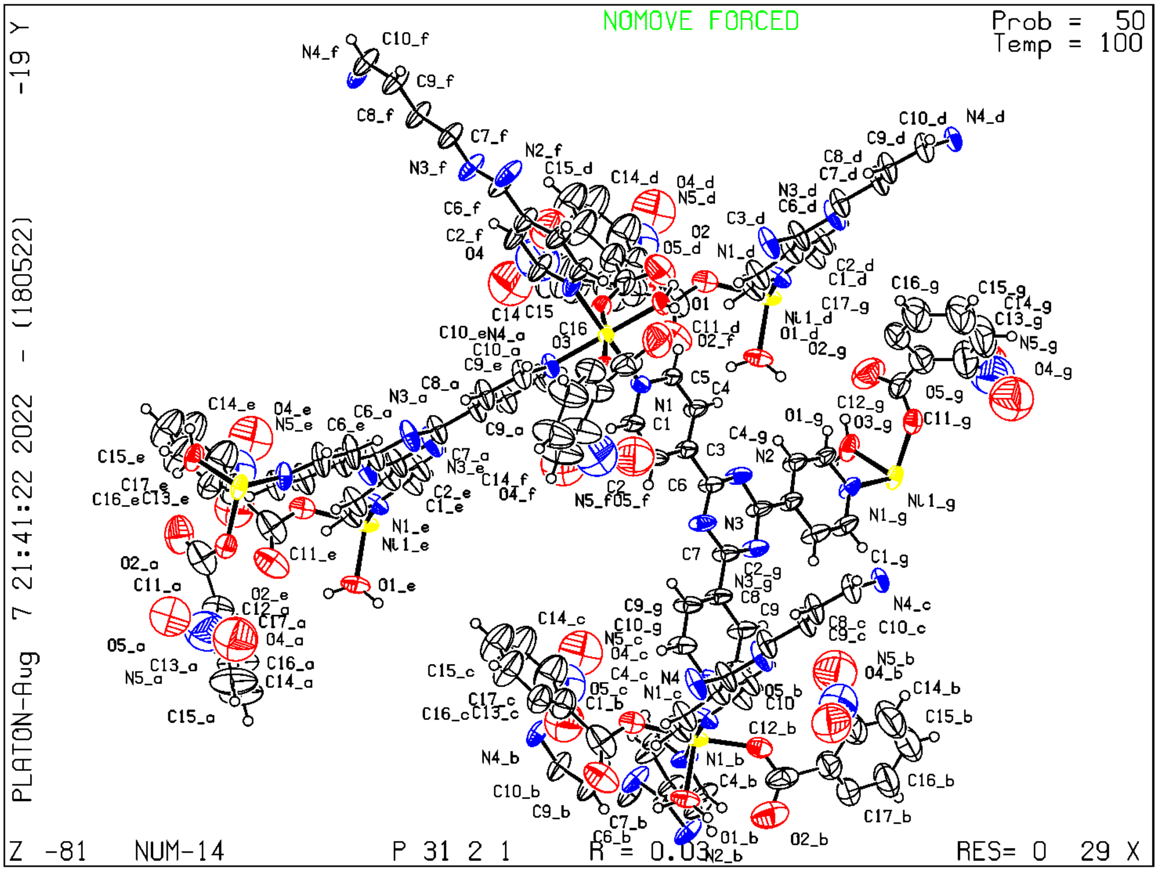

Supplement: Supplementary file 1 [file molecules-27-05929-s001.zip › NUM-14_checkcif.pdf]
