# Supplementary material for: Customizing Pore System in a Microporous Metal–Organic Framework for Efficient C2H2 Separation from CO2 and C2H4
Source: Molecules. 2022 Sep 12;27(18):5929. doi: 10.3390/molecules27185929 (PMC9502222; doi:10.3390/molecules27185929)
Supplement: Supplementary file 1 [file molecules-27-05929-s001.zip › Supporting information.pdf]

Supporting Information

Customizing Pore System in a Microporous Metal-Organic Framework for Efficient C_2H_2 Separation from CO_2 and C_2H_4

Qiang Zhang†, Guan-Nan Han†, Xin Lian, Shan-QingYang, Tong-Liang Hu*

School of Materials Science and Engineering, National Institute for Advanced Materials, Nankai University, Tianjin 300350, China.

* Correspondence: tlhu@nankai.edu.cn (T.-L. Hu)

† These authors contributed equally to this work.

Table S1. Crystal data and structure refinement parameters for **NUM-14**

Empirical formula	$C_{26}H_{17}N_7NiO_7$
Formula weight (g mol^{-1})	607.82
Crystal system	trigonal
Space group	$P3_121$
a (Å)	15.11740(1)
b (Å)	15.11740(1)
c (Å)	18.8520(2)
α(°)	90
β(°)	90
γ(°)	120
V (Å^3)	3731.14(6)
Z	3
ρ_{calc} (g cm^{-3})	0.812
μ (mm^{-1})	0.849
F (000)	933.0
2Θ range for data collection/°	6.752 to 147.228
Index ranges	$-18 \le h \le 15$, $-13 \le k \le 18$, $-23 \le l \le 17$
Reflections collected	12400
Independent reflections	4868 [R_{int} = 0.0206, R_{sigma} = 0.0236]
Goodness-of-fit on F^2	1.075
Final R indexes [I>=2σ (I)][a]	R_1 = 0.0339, wR_2 = 0.0967
Final R indexes [all data][b]	R_1 = 0.0354, wR_2 = 0.0991
Largest diff. peak/hole/e Å^{-3}	0.21/-0.21
CCDC deposition number	2163045

[a]$R_1=\sum||F_o| - |F_c||/\sum|F_o|$, [b]$wR_2=\{\sum[w(F_o^2 - F_c^2)^2]/\sum w(F_o^2)^2\}^{1/2}$

Table S2. Comparisons of C_2H_2 uptake and selectivities of C_2H_2/CO_2 and C_2H_2/C_2H_4 for **NUM-14a** and other MOFs

**MOFs**	**C_2H_2 uptake mmol g^{-1}**	**C_2H_2/CO_2 (50:50, v/v) selectivity**	**Ref.**
CuI@UiO-66-$(COOH)_2$	2.14	185.00	[1]
ATC-Cu	5.01	53.60	[2]
MOF-OH	3.04	25.00	[3]
IPM-101	3.04	12.3	[4]
FJU-118a	3.96	7.80	[5]
BUT-85	2.95	6.10	[6]
[Ca(dtztp)$_{0.5}$]	4.91	1.70	[7]
[Ni(tzba)$_{0.5}$(F)(bpy)]	5.58	2.20	[8]
CAU-10H	5.13	2.50	[9]
SNNU-5-Sc	5.13	2.66	[10]
**NUM-14a**	4.44	3.37	**This work**
**MOFs**	**C_2H_2 uptake mmol g^{-1}**	**C_2H_2/C_2H_4 (1:99, v/v) selectivity**	**Ref.**
NUM-12a	4.42	1.13	[11]
Zn(ad)(int)	-	1.61	[12]
ZJNU-14	4.00	1.59	[13]
ZJNU-7	5.04	1.77	[14]
UiO-67-$(NH_2)_2$	5.90	2.1	[15]
**NUM-14a**	4.44	1.63	**This work**

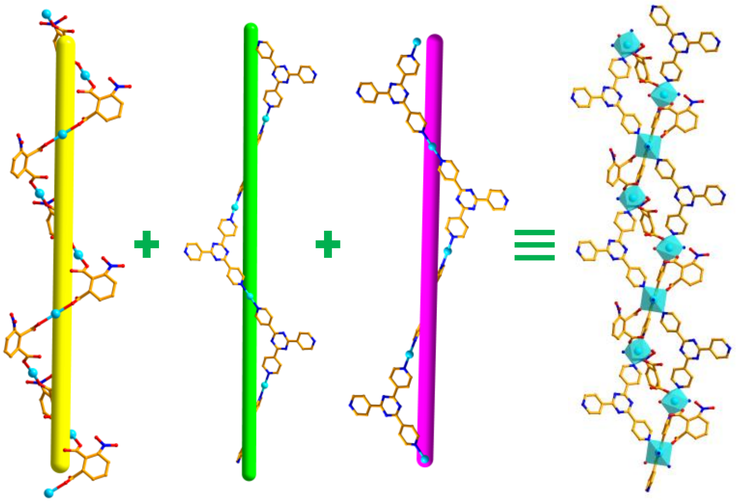

Figure S1. Three kinds of helix chains constitute the channel column of **NUM-14**.

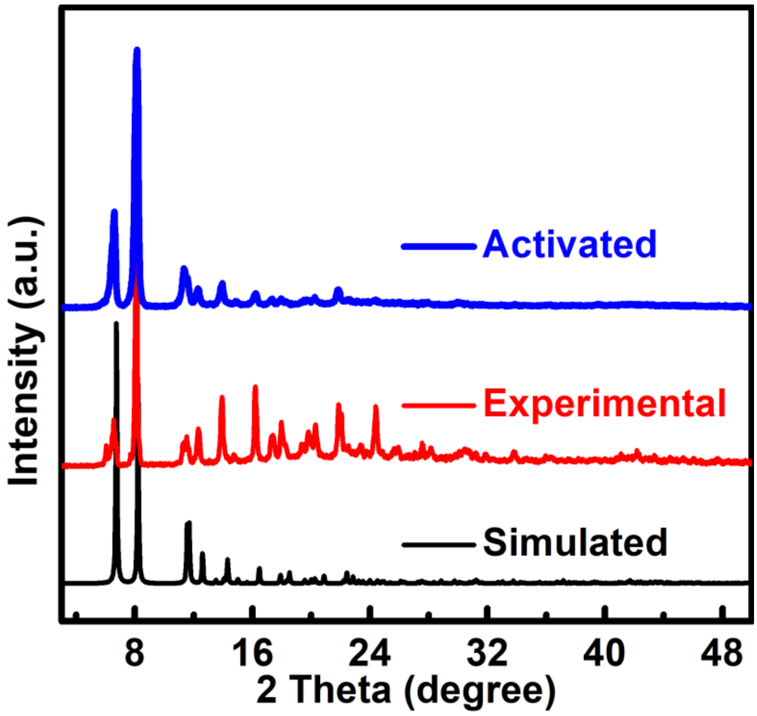

Figure S2. Comparison of simulated, experimental, and activated PXRD patterns of **NUM-14**.

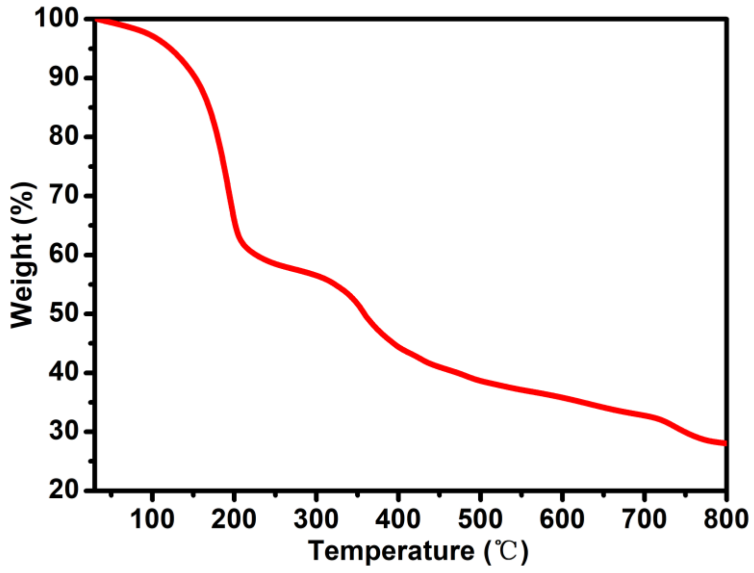

Figure S3. TGA curve for **NUM-14** under Ar atmosphere.

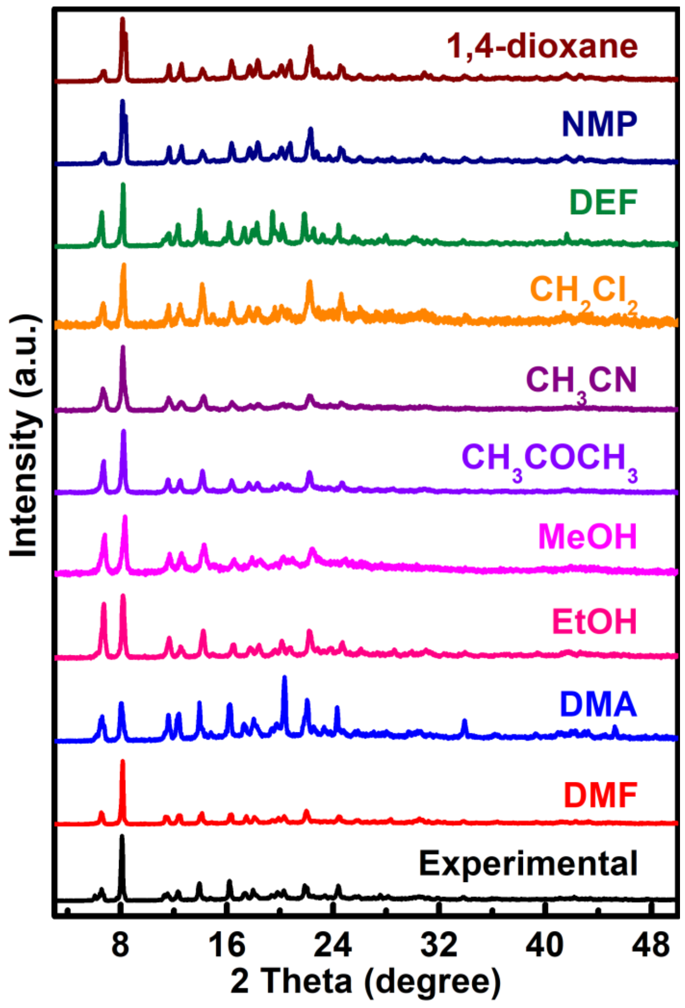

Figure S4. The PXRD patterns for **NUM-14** after immersed in common solvents a week.

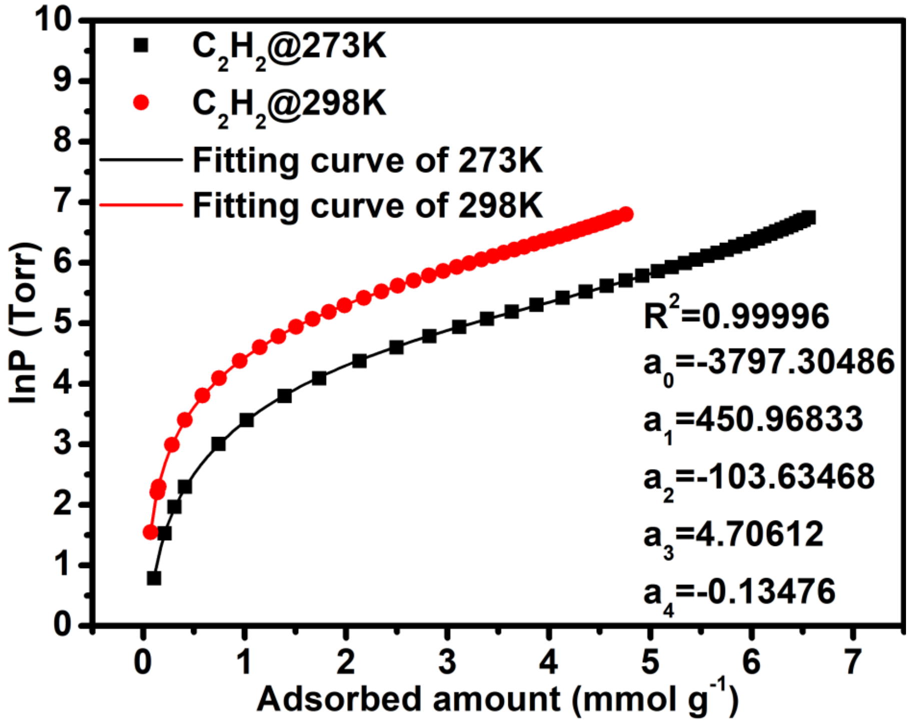

Figure S5. The details of Virial equation (solid lines) fitting to the experimental C_2H_2 adsorption data (symbols) for **NUM-14a**.

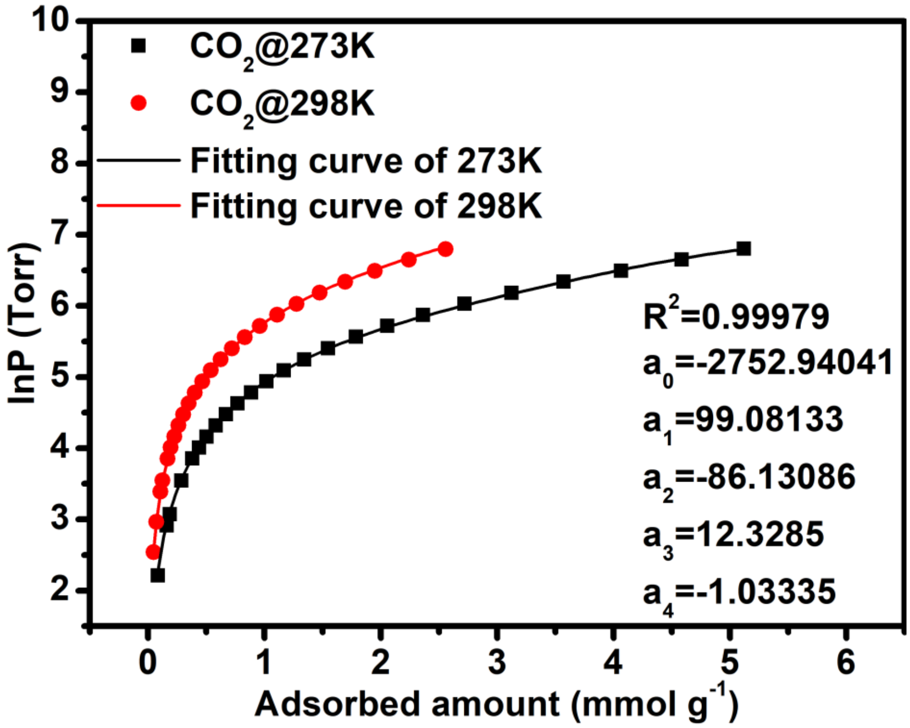

Figure S6. The details of Virial equation (solid lines) fitting to the experimental CO_2 adsorption data (symbols) for **NUM-14a**.

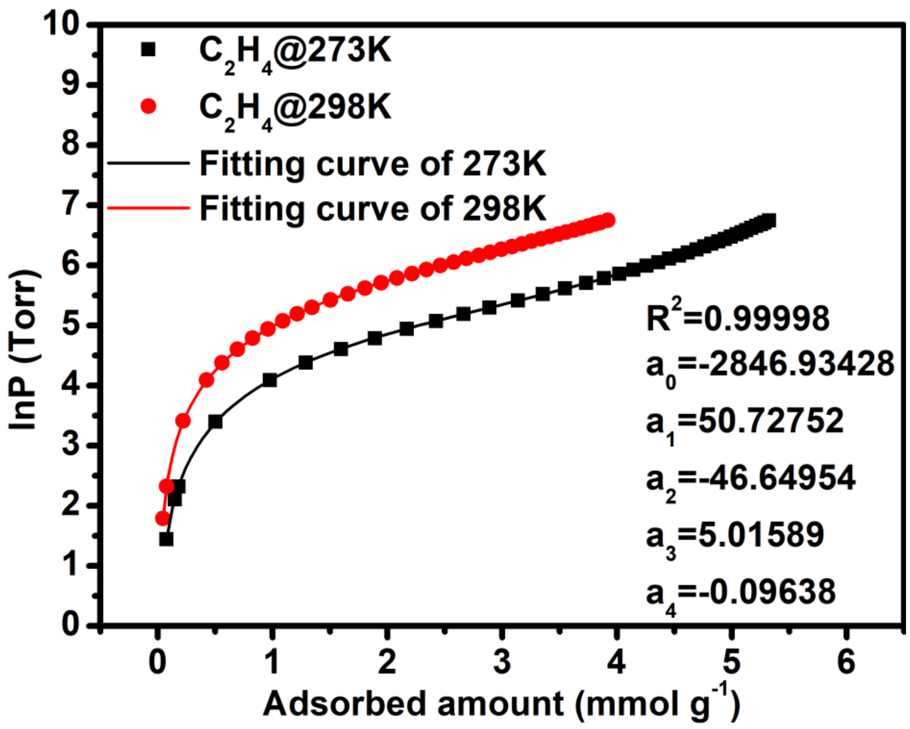

Figure S7. The details of Virial equation (solid lines) fitting to the experimental C_2H_4 adsorption data (symbols) for **NUM-14a**.

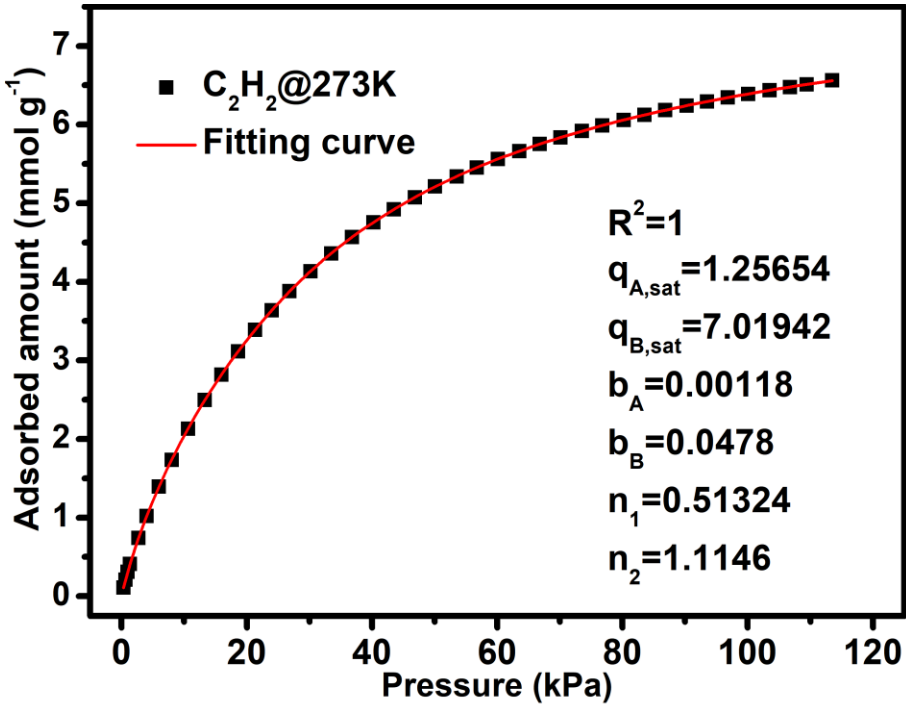

Figure S8. The details of dual-site Langmuir-Freundlich isotherm (solid lines) fitting to the experimental C_2H_2 adsorption data (symbols) for **NUM-14a** at 273 K.

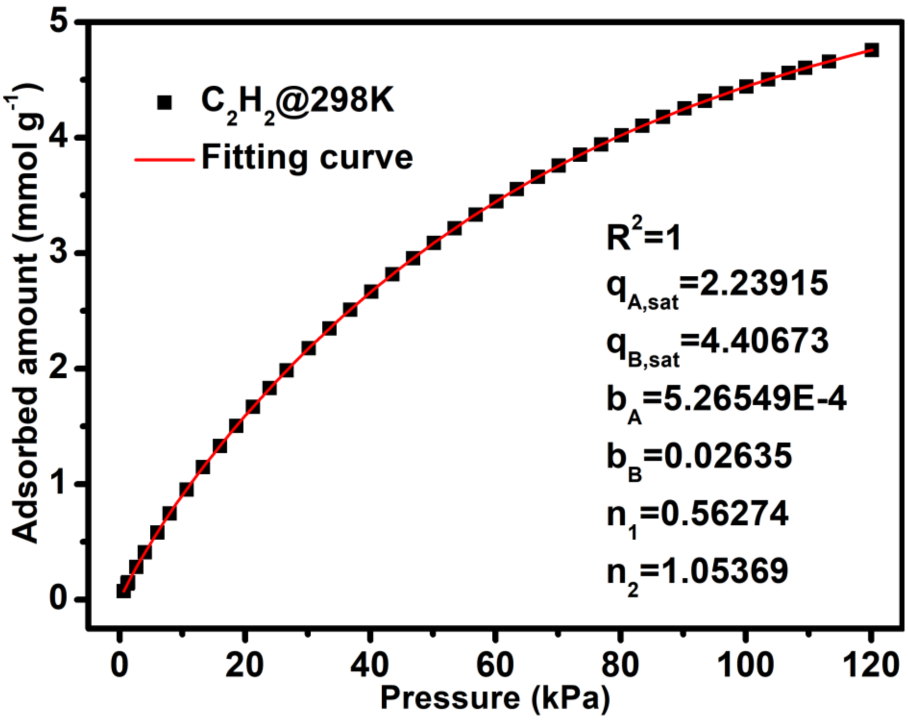

Figure S9. The details of dual-site Langmuir-Freundlich isotherm (solid lines) fitting to the experimental C_2H_2 adsorption data (symbols) for **NUM-14a** at 298 K.

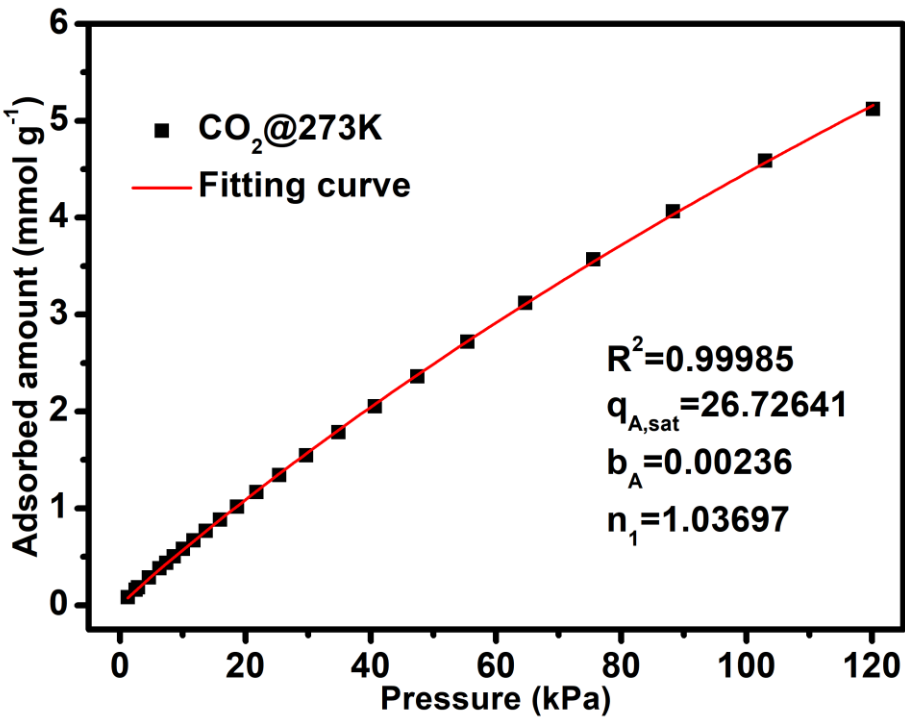

Figure S10. The details of single-site Langmuir-Freundlich isotherm (solid lines) fitting to the experimental CO_2 adsorption data (symbols) for **NUM-14a** at 273 K.

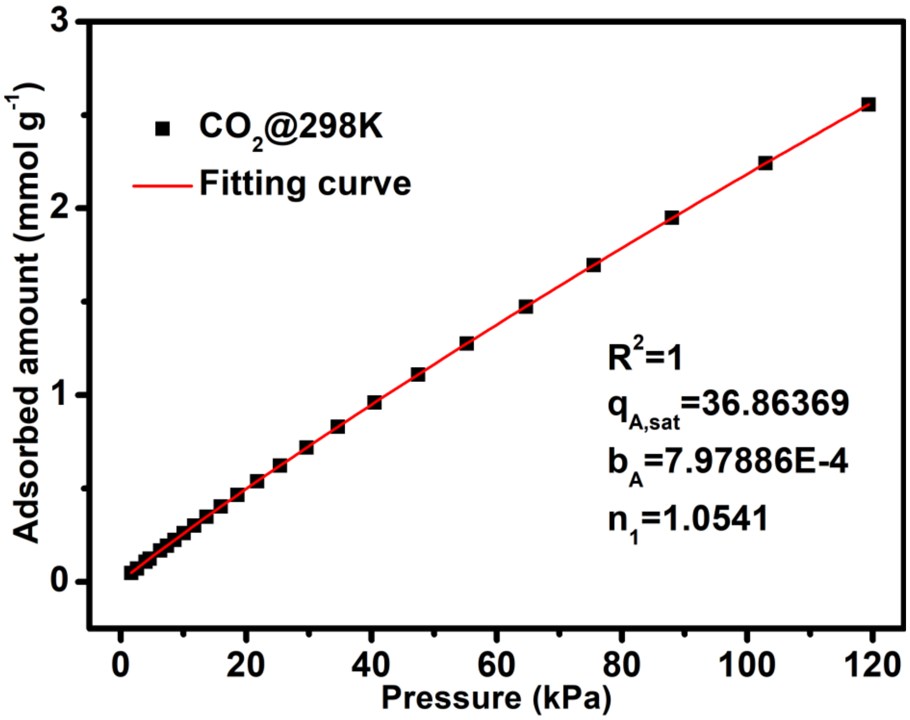

Figure S11. The details of single-site Langmuir-Freundlich isotherm (solid lines) fitting to the experimental CO_2 adsorption data (symbols) for **NUM-14a** at 298 K.

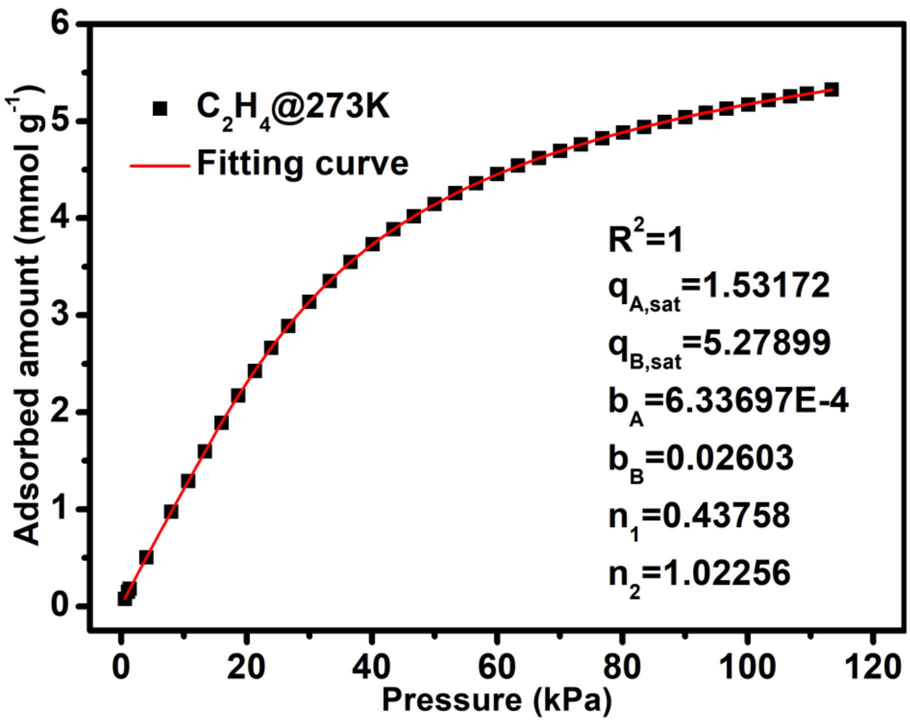

Figure S12. The details of dual-site Langmuir-Freundlich isotherm (solid lines) fitting to the experimental C_2H_4 adsorption data (symbols) for **NUM-14a** at 273 K.

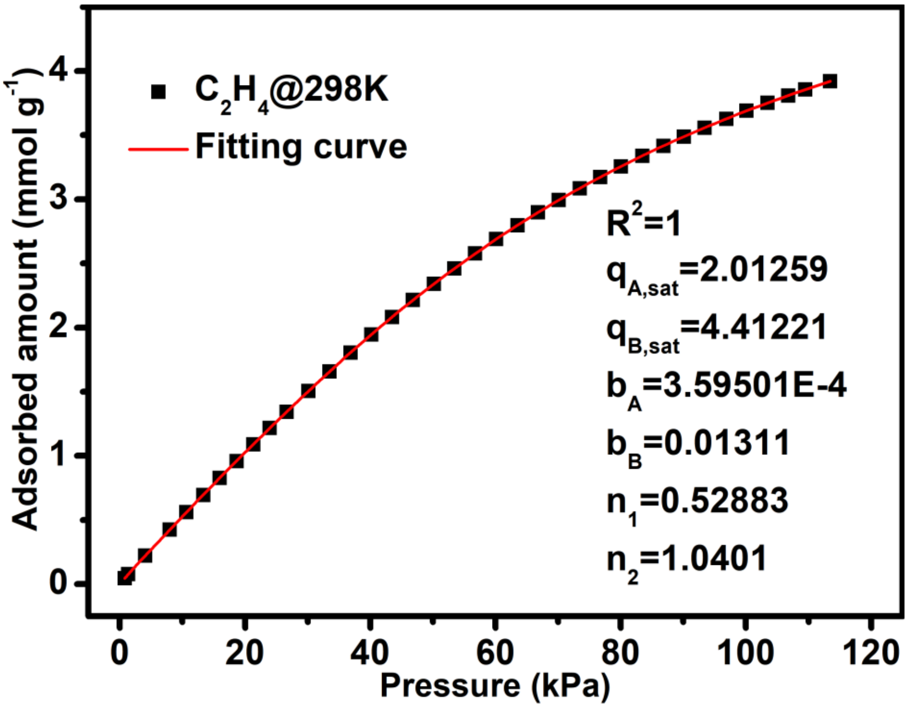

Figure S13. The details of dual-site Langmuir-Freundlich isotherm (solid lines) fitting to the experimental C_2H_4 adsorption data (symbols) for **NUM-14a** at 298 K.

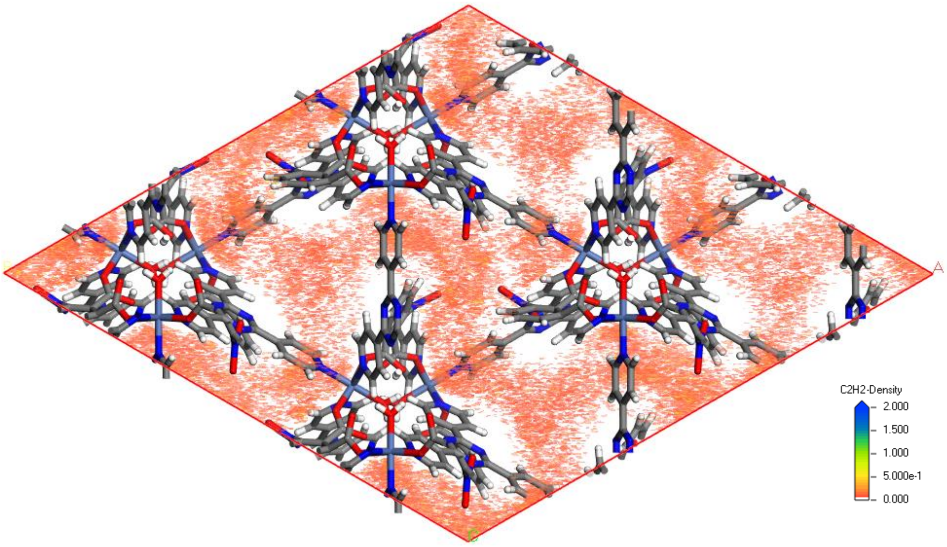

Figure S14. Density distribution of C_2H_2 in **NUM-14a** at 298 K and 1 bar.

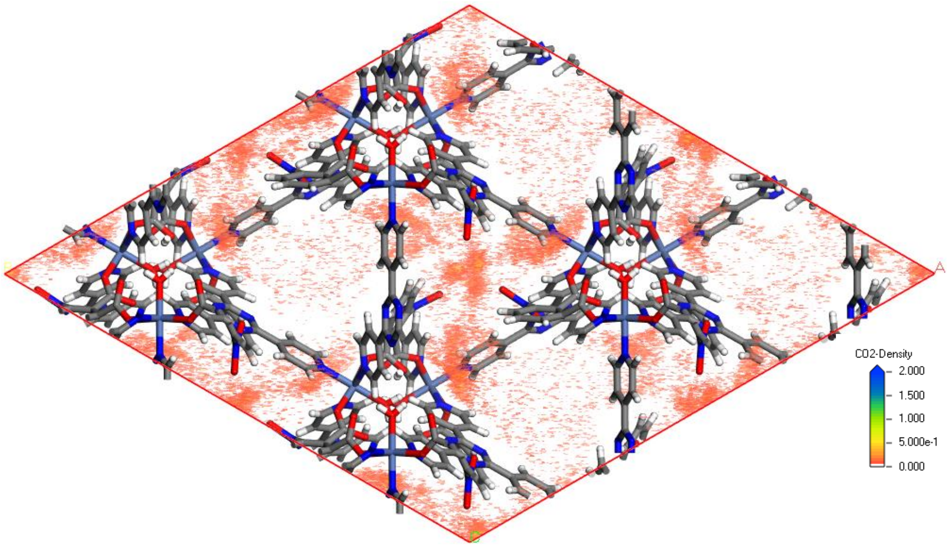

Figure S15. Density distribution of CO_2 in **NUM-14a** at 298 K and 1 bar.

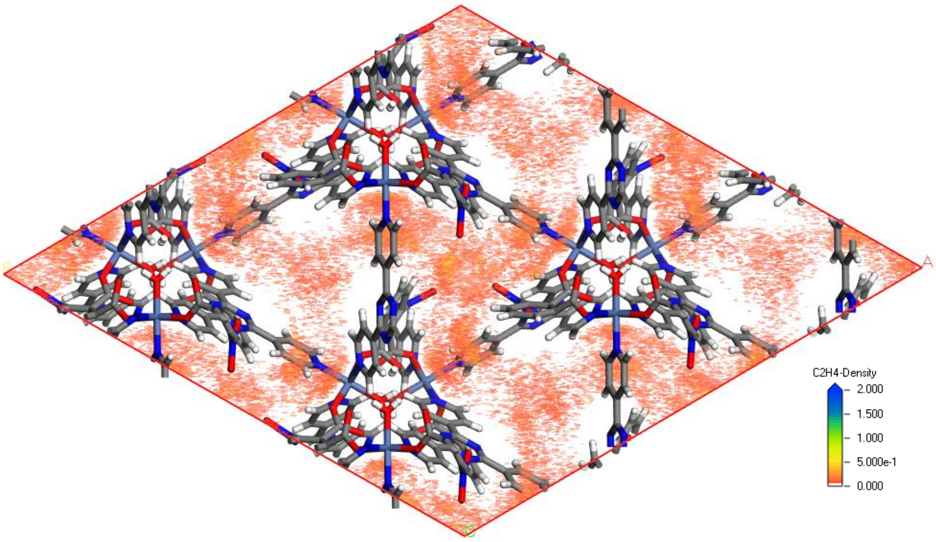

Figure S16. Density distribution of C_2H_4 in **NUM-14a** at 298 K and 1 bar.

References

1. Zhang, L.; Jiang, K.; Yang, L.; Li, L.; Hu, E.; Yang, L.; Shao, K.; Xing, H.; Cui, Y.; Yang, Y.; Li, B.; Chen, B.; Qian, G. Benchmark C_2H_2/CO_2 separation in an ultra-microporous metal-organic framework via copper(I)-alkynyl chemistry. *Angew. Chem. Int. Ed.* **2021**, *60, 15995-16002.*
2. Niu, Z.; Cui, X.; Pham, T.; Verma, G.; Lan, P. C.; Shan, C.; Xing, H.; Forrest, K. A.; Suepaul, S.; Space, B.; Nafady, A.; Al-Enizi, A. M.; Ma, S., A MOF-based ultra-strong acetylene nano-trap for highly efficient C_2H_2/CO_2 separation. *Angew. Chem. Int. Ed.* **2021**, *60, 5283-5288.*
3. Gong, W.; Cui, H.; Xie, Y.; Li, Y.; Tang, X.; Liu, Y.; Cui, Y.; Chen, B., Efficient C_2H_2/CO_2 separation in ultramicroporous metal-organic frameworks with record C_2H_2 storage density. *J. Am. Chem. Soc.* **2021**, *143, 14869-14876.*
4. Sharma, S.; Mukherjee, S.; Desai, A. V.; Vandichel, M.; Dam, G. K.; Jadhav, A.; Kociok-Köhn, G.; Zaworotko, M. J.; Ghosh, S. K., Efficient capture of trace acetylene by an ultramicroporous metal-organic framework with purine binding sites. *Chem. Mater. 2021,* **33**, *5800-5808.*
5. Song, Q.; Yang, Y.; Yuan, F.; Zhu, S.; Wang, J.; Xiang, S.; Zhang, Z., Electrostatic force-driven lattice water bridging to stabilize a partially charged indium MOF for efficient separation of C_2H_2/CO_2 mixtures. *J. Mater. Chem. A* **2022**, *10, 9363-9369.*
6. Si, G.-R.; Wu, W.; He, T.; Xu, Z.-C.; Wang, K.; Li, J.-R., Stable Bimetallic Metal-organic framework with dual-functional pyrazolate-carboxylate ligand: Rational construction and C_2H_2/CO_2 separation. *ACS Materials Letters* **2022**, *4, 1032-1036.*
7. Wang, G.-D.; Li, Y.-Z.; Zhang, W.-F.; Hou, L.; Wang, Y.-Y.; Zhu, Z., Acetylene separation by a Ca-MOF containing accessible sites of open metal centers and organic groups. *ACS Appl. Mater. Interfaces* **2021**, *13, 58862-58870.*
8. Wang, G.-D.; Wang, H.-H.; Shi, W.-J.; Hou, L.; Wang, Y.-Y.; Zhu, Z., A highly stable MOF with F and N accessible sites for efficient capture and separation of acetylene from ternary mixtures. *J. Mater. Chem. A* **2021**, *9, 24495-24502.*
9. Ye, Y.; Xian, S.; Cui, H.; Tan, K.; Gong, L.; Liang, B.; Pham, T.; Pandey, H.; Krishna, R.; Lan, P. C.; Forrest, K. A.; Space, B.; Thonhauser, T.; Li, J.; Ma, S., Metal-organic framework based hydrogen-bonding nanotrap for efficient acetylene storage and separation. *J. Am. Chem. Soc.* **2022**, *144, 1681-1689.*
10. Lv, H.-J.; Zhang, J.-W.; Jiang, Y.-C.; Li, S.-N.; Hu, M.-C.; Zhai, Q.-G., Micropore regulation in ultrastable $[Sc3O]^-$ organic frameworks for acetylene storage and purification. *Inorg. Chem.* **2022**, *61, 3553-3562.*
11. Zhang, Q.; Yang, S.-Q.; Zhou, L.; Yu, L.; Li, Z.-F.; Zhai, Y.-j.; Hu, T.-L., Pore-space partition through an embedding metal-carboxylate chain-induced topology upgrade strategy for the separation of acetylene/ethylene. *Inorg. Chem.* **2021**, *60, 19328-19335.*
12. Ding, Q.; Zhang, Z.; Liu, Y.; Chai, K.; Krishna, R.; Zhang, S. One-step ethylene purification from ternary mixtures in a metal-organic framework with customized pore chemistry and shape. *Angew. Chem. Int. Ed.* **2022**, *DOI: 10.1002/anie.202208134.*
13. Jiang, Z.; Fan, L.; Zhou, P.; Xu, T.; Chen, J.; Hu, S.; Chen, D.-L.; He, Y., An N-oxide-functionalized nanocage-based copper-tricarboxylate framework for the selective capture of C_2H_2. *Dalton Transactions* **2020**, *49, 15672-15681.*
14. Jiang, Z.; Fan, L.; Zhou, P.; Xu, T.; Hu, S.; Chen, J.; Chen, D.-L.; He, Y., An aromatic-rich cage-based MOF with inorganic chloride ions decorating the pore surface displaying the preferential adsorption of C_2H_2 and C_2H_6 over C_2H_4. *Inorg. Chem. Front.* **2021**, *8, 1243-1252.*

15. Gu, X.-W.; Wang, J.-X.; Wu, E.; Wu, H.; Zhou, W.; Qian, G.; Chen, B.; Li, B., Immobilization of lewis basic sites into a stable ethane-selective MOF enabling one-step separation of ethylene from a ternary mixture. *J. Am. Chem. Soc.* **2022**, *144, 2614-2623*.
